# Screening for Health-Related Social Needs and Collaboration With External Partners Among US Hospitals

**DOI:** 10.1001/jamanetworkopen.2023.30228

**Published:** 2023-08-23

**Authors:** Jason J. Ashe, Matthew C. Baker, Carla S. Alvarado, Philip M. Alberti

**Affiliations:** 1 Association of American Medical Colleges, Washington, District of Columbia

## Abstract

**Importance:**

In recent years, hospitals and health systems have reported increasing rates of screening for patients’ individual and community social needs, but few studies have explored the national landscape of screening and interventions directed at addressing health-related social needs (HRSNs) and social determinants of health (SDOH).

**Objective:**

To evaluate the associations of hospital characteristics and area-level socioeconomic indicators to quantify the presence and intensity of hospitals’ screening practices, interventions, and collaborative external partnerships that seek to measure and ameliorate patients’ HRSNs and SDOH.

**Design, Setting, and Participants:**

This cross-sectional study used national data from the American Hospital Association Annual Survey Database for fiscal year 2020. General-service, acute-care, nonfederal hospitals were included in the study’s final sample, representing nationally diverse hospital settings. Data were analyzed from July 2022 to February 2023.

**Exposures:**

Organizational characteristics and area-level socioeconomic indicators.

**Main Outcomes and Measures:**

The outcomes of interest were hospital-reported patient screening of and strategies to address 8 HRSNs and 14 external partnership types to address SDOH. Composite scores for screening practices and external partnership types were calculated, and ordinary least-square regression analyses tested associations of organizational characteristics with outcome measures.

**Results:**

Of 2858 US hospital respondents (response rate, 67.0%), most hospitals (79.2%; 95% CI, 77.7%-80.7%) reported screening patients for at least 1 HRSN, with food insecurity or hunger needs (66.1%; 95% CI, 64.3%-67.8%) and interpersonal violence (66.4%; 95% CI, 64.7%-68.1%) being the most commonly screened social needs. Most hospitals (79.4%; 95% CI, 66.3%-69.7%) reported having strategies and programs to address patients’ HRSNs; notably, most hospitals (52.8%; 95% CI, 51.0%-54.5%) had interventions for transportation barriers. Hospitals reported a mean of 4.03 (95% CI, 3.85-4.20) external partnership types to address SDOH and 5.69 (5.50-5.88) partnership types to address HRSNs, with local or state public health departments and health care practitioners outside of the health system being the most common. Hospitals with accountable care contracts (ACCs) and bundled payment programs (BPPs) reported higher screening practices (ACC: β = 1.03; SE = 0.13; BPP: β = 0.72; SE = 0.14), interventions (ACC: β = 1.45; SE = 0.12; BPP: β = 0.61; SE = 0.13), and external partnership types to address HRSNs (ACC: β = 2.07; SE = 0.23; BPP: β = 1.47; SE = 0.24) and SDOH (ACC: β = 2.64; SE = 0.20; BPP: β = 1.57; SE = 0.21). Compared with nonteaching, government-owned, and for-profit hospitals, teaching and nonprofit hospitals were also more likely to report more HRSN-directed activities. Patterns based on geographic and area-level socioeconomic indicators did not emerge.

**Conclusions and Relevance:**

This cross-sectional study found that most US hospitals were screening patients for multiple HRSNs. Active participation in value-based care, teaching hospital status, and nonprofit status were the characteristics most consistently associated with greater overall screening activities and number of related partnership types. These results support previously posited associations about which types of hospitals were leading screening uptake and reinforce understanding of the role of hospital incentives in supporting health equity efforts.

## Key Points

**Question:**

What hospital organizational characteristics and factors are associated with screening for and addressing patients’ health-related social needs and community-level social determinants of health?

**Findings:**

In this cross-sectional study of 2858 US hospitals, most hospitals screened patients for and had interventions to address at least 1 health-related social need and reported at least 1 partnership to support related activities. Participation in value-based care, including accountable care contracts and bundled payments, was associated with more screening efforts, strategies to address needs and social determinants of health, and external partnership types to support interventions.

**Meaning:**

These findings suggest that hospitals’ policy-related organizational decisions hold the promise of progress toward equitable health care delivery.

## Teaser

This cross-sectional study evaluates the associations of hospital characteristics with programs and strategies to screen for and address health-related social needs and social determinants of health.

## Introduction

It is now widely recognized in medicine that a person’s health is largely determined by social factors, stress exposure, and the environment in which they live, comprising 30% to 55% of health outcomes.^1,2,3,4^ The impacts of these social factors have led US hospitals to recognize that effective medical treatment requires attending to patients’ immediate nonmedical needs. These health impacts are commonly divided into 2 levels: health-related social needs (HRSNs) at the individual level and social determinants of health (SDOH) at the societal, community, or population level. While HRSNs, such as food insecurity, transportation access, employment status, and education, are the most proximal to hospitals and urgent for treatment and continuity of care, the broader SDOH comprise “non-medical factors that influence health outcomes,” including “the conditions in which people are born, grow, live, and age,”^4^ which are created and recreated by societal values that are operationalized by institutions via policies that allocate resources at local, state, and national levels.

Burgeoning pressures to shift health services from a payment-based system to value-based care (VBC) have prompted organizational interventions targeting patient population health,^5,6^ including HRSN screening and referrals to local partners or community-based organization to address related concerns or barriers.^7^ Hospitals, health care systems, and institutions are addressing both HRSNs^8,9^ and SDOH.^10^ Based on recent studies reporting that addressing HRSNs is associated with improved patient satisfaction, overall health, and quality of life, as well as with reduced unnecessary patient spending, such as readmission and urgent or emergency care visits^11,12,13,14^ that use hospital resources when they may already be near capacity. Hospitals and health systems have implemented numerous activities to address HRSNs. These include temporary housing vouchers, ridesharing or financial assistance to cover public transportation fares, and access to food pantries to provide short-term alleviation for patients at high risk of adverse outcomes who present with multiple HRSNs.^15,16,17^ Hospitals have identified HRSNs as a priority, and many have allocated the will and resources to design, develop, and deliver population-level interventions.^3,7,9,18,19,20^

Although some studies have examined the prevalence of screening practices,^8^ information on hospital organizational characteristics associated with these practices is nascent.^21^ This study leverages national hospital survey data to identify which organizational characteristics and financial arrangements are associated with HRSN screening practices, the internal strategies and programs developed to address them, and external partnerships developed to address the greater context of SDOH.

## Methods

### Data Collection

This cross-sectional study was determined to be exempt from review and informed consent by the institutional review board of the Association of American Medical Colleges because it was not human participants research. This study adhered to the Strengthening the Reporting of Observational Studies in Epidemiology (STROBE) reporting guideline.

We used the fiscal year (FY) 2020 edition of the American Hospital Association Annual Survey Database, a voluntary national survey released in November 2021 and collected continuously after the close of each hospital’s FY 2020. The Annual Survey Database collects information from leadership across all US hospitals on hospital characteristics, including ownership, hospital bed size, geographic location, participation in VBC programs, as well as items added in FY 2020 assessing screening practices and activities related to addressing HRSNs and SDOH. For this study, we narrowed the 6165 national hospitals to 4265 acute care, general service, nonfederal hospitals in the US (excluding territories). Among these hospitals, 2858 (67% response rate) had available, valid survey responses for the section addressing screening and partnerships. We used supplementary data from the Robert Graham Center Social Deprivation Index (2015-2019) and County Health Rankings (2020) to characterize socioeconomic disadvantage in areas surrounding hospital locations.

### Outcome Measures

We constructed 4 outcomes by counting the number of HRSNs or partnership types: (1) the number of HRSNs the hospital reported screening (range, 0-8), (2) the number of HRSNs the hospital reported having programs or strategies to address (range, 0-8), (3) the number of reported partnership types to meet patients’ HRSNs (range, 0-14), and (4) the number of reported partnership types to meet community-level SDOH (range, 0-14). Details on the survey’s question and response options are presented in eAppendix 1 in Supplement 1.

### Organizational Characteristics

We used 100-bed ranges to categorize hospital size. We classified hospitals into the 9 US Census divisions based on the hospital’s state location. Urbanicity (metropolitan, micropolitan, or rural) reflected hospitals’ Core-Based Statistical Area location. As a proxy for a hospital’s financial health, we used total all-payer financial margins calculated from FY 2019 revenue and costs reported to Medicare via the Healthcare Cost Report Information System, using the October 2021 release. We constructed 2 separate and dichotomous variables to account for reported participation in VBC programs among survey respondents: 1 variable for a hospital’s active enrollment in accountable care contracts in any of the following 4 plans: Medicaid, traditional Medicare, Medicare Advantage plan, or commercial insurance plan, and 1 variable for participation in bundled payment programs in any of the same 4 plans. Medicaid discharge ratio was defined as the percentage of nonunique discharged Medicaid patients over the total number of admitted patients. Major teaching hospitals are defined as having a teaching intensity or intern-and-resident–to-bed ratio of greater than or equal to 0.25 in Medicare’s Inpatient Prospective Payment System FY 2022 final rule impact file, while minor teaching hospitals are those with intern-and-resident–to-bed ratios less than 0.25 but greater than 0.

Because hospitals operate in the context of their communities, we included additional community-level measures related to HRSNs and SDOH. We included the Social Deprivation Index from the Robert Graham Center, an area-level measure based on 7 characteristics that quantify variations in health outcomes (eg, percentage of population living in poverty, percentage of population with >12 years education, overcrowded housing), originally from the American Community Survey from 2015 to 2019 (range, 1-100; higher score indicates greater area-level deprivation).^22^ The 2020 County Health Rankings database provided the percentage of uninsured adults younger than 65 years by county, based on 2017 measurement periods.^23^

### Statistical Analysis

Ordinary least-square regression analyses assessed associations of hospital characteristics with the 4 specified outcome variables for screening and partnerships. All 4 regression base models include as covariates the complete set of hospital and community characteristics. For all analyses, we assigned reference groups as the categories with the largest number of hospitals represented in the final sample. For each of the 4 outcomes, we excluded hospitals with missing data from analyses. We also specify several additional versions of these regressions to test for robustness (eAppendix 2 in Supplement 1).

To address potential nonresponse bias in ensuring the sample’s national representativeness, we applied sampling weights throughout all analyses for each hospital according to their inverse probability of survey response. We calculated sample weights by the standard method as the inverse of the projected probability of responding to the survey. Projected response probability for each hospital was estimated with a logistic regression model equalizing respondent and nonrespondent characteristics across hospital bed size; ownership, tax-exempt, and teaching statuses; and geographic location. Throughout all our models, we determined significance using conventional, 2-sided levels of α, *P* = .05. We performed all statistical calculations and analyses using SAS software version 9.4 (SAS Institute). Data were analyzed from July 2022 to February 2023.

## Results

### Descriptive Analyses

A total of 2858 general-service, acute-care, nonfederal hospitals were included in the study’s final sample, representing nationally diverse hospital settings. Hospitals reported a weighted mean (SD) of 18.11% (14.48%) discharges among Medicaid patients; the mean (SD) proportion of uninsured adults aged younger than 65 years was 10.61% (6.10%), and the weighted mean (SD) Social Deprivation Index was 44.52 (33.22). The composition of the sample is presented in Table 1. Most hospitals reported screening for (2301 hospitals; weighted, 79.2%; 95% CI, 77.7%-80.7%) and having a strategy or program to address (2324 hospitals; weighted, 79.4%; 95% CI, 77.9%-80.9%) HRSNs (Table 2). Screening and addressing often, but not always, cooccurred for a given HRSN (Table 2).

**Table 1.** Characteristics of Hospitals in the Final Sample

Characteristic	Hospitals, No. (N = 2858)^a^	Weighted proportion of all hospitals	Proportion screening for and addressing ≥1 HRSN
Bed capacity, No.^b^			
<100	1367	0.52	0.60
100-199	547	0.20	0.75
200-299	332	0.11	0.82
300-399	220	0.07	0.83
400-499	127	0.04	0.85
≥500	265	0.07	0.88
Regions^c^			
New England	118	0.04	0.85
Mid-Atlantic	272	0.08	0.86
South Atlantic	362	0.15	0.72
East North Central	494	0.16	0.79
East South Central	124	0.09	0.68
West North Central	489	0.15	0.61
West South Central	522	0.14	0.59
Mountain	218	0.08	0.60
Pacific	259	0.11	0.71
Teaching status^d^			
Major teaching hospitals	262	0.07	0.90
Other teaching hospitals	603	0.18	0.80
Nonteaching hospitals	1993	0.74	0.65
Tax-exempt status^b^			
Government	561	0.22	0.58
For-profit	272	0.14	0.49
Nonprofit	2025	0.64	0.79
Urbanicity^e^			
Metropolitan	1760	0.58	0.77
Micropolitan	494	0.17	0.71
Rural	604	0.25	0.53
Accountable care contract^b^			
Any payer	912	0.28	0.89
Traditional Medicare	753	0.23	0.89
Medicare Advantage plan	487	0.15	0.93
Commercial insurance plan	607	0.18	0.92
Medicaid	270	0.08	0.92
Bundled payment program^b^			
Any payer	828	0.26	0.84
Traditional Medicare	721	0.23	0.85
Medicare Advantage plan	175	0.05	0.90
Commercial insurance plan	302	0.09	0.87
Medicaid	91	0.03	0.84

Abbreviation: HRSN, health-related social need.

^a^ Unweighted number of survey respondents per category.

^b^ Data from the American Hospitals Association’s Annual Survey Database fiscal year 2020.

^c^ Regional differences were defined using US Census divisions and state codes.

^d^ Teaching status was defined by intern-and-resident–to-bed ratio in Medicare’s Inpatient Prospective Payment System fiscal year 2022 final rule impact file.

^e^ Urbanicity defined by the Census Bureau’s urban-rural classification system.

**Table 2.** Hospital HRSN Screening Practices and Strategies to Address HRSNs (N = 2858 Hospitals)

Measure	Hospitals, weighted % (95% CI)		
	With screening for HRSN	With strategies to address HRSNs	Screening and with strategies to address HRSNs
Domains, No.			
≥1	79.2 (77.7-80.7)	79.4 (77.9-80.9)	59.8 (68.2-71.5)
≥4	63.1 (61.3-64.9)	52.9 (51.1-54.8)	45.4 (43.5-47.2)
8	29.4 (27.7-31.0)	21.6 (20.1-23.1)	17.4 (16.0-18.8)
Specific domain addressed			
Food insecurity or hunger	66.1 (64.3-67.8)	61.9 (60.1-63.7)	55.3 (53.5-57.1)
Transportation	64.8 (63.1-66.6)	51.5 (49.6-53.3)	55.1 (53.3-56.9)
Interpersonal violence	66.4 (64.7-68.1)	64.5 (62.8-66.3)	48.1 (46.3-50.0)
Social isolation	62.9 (61.1-64.7)	47.9 (46.0-49.7)	44.6 (42.8-46.4)
Housing	64.6 (62.9-66.4)	49.9 (48.1-51.8)	43.9 (42.0-45.7)
Utilities	47.4 (45.6-49.2)	40.9 (39.1-42.7)	33.3 (31.6-35.0)
Employment or income	45.7 (43.8-47.5)	38.9 (37.2-40.7)	29.4 (27.7-31.1)
Education	39.9 (38.1-41.7)	35.9 (34.1-37.6)	28.7 (27.1-30.4)
Total HRSNs, mean (95% CI), No. (range, 0-8)	4.58 (4.46-4.69)	3.91 (3.80-4.02)	3.38 (3.27-3.50)

Abbreviation: HRSN, health-related social need.

More than half of hospitals reported at least 1 external partnership type to address HRSNs (1971 hospitals; weighted, 68.0%; 95% CI, 66.3%-69.7%) and SDOH vis-à-vis community-level health initiatives (1603 hospitals; weighted, 53.7%; 95% CI, 51.8%-55.5%). Screening rates and the number of strategies and programs varied by HRSN, with most hospitals screening for interpersonal violence (weighted, 66.4%; 95% CI, 64.7%-68.1%) and food insecurity (weighted, 66.1%; 95% CI, 64.3%-67.8%); and many reporting interventions for transportation barriers (weighted, 52.8%; 95% CI, 51.0%-54.5%) (Table 2). There was also a broad scope of external partnership types to address HRSNs and SDOH. Most hospitals that reported any external partnership types reported relationships with health care practitioners outside of their hospital or health system to address individual-level HRSNs and with local or state public health departments to address community-level SDOH, respectively (Table 3).

**Table 3.** External Partnerships to Meet Patients’ HRSNs and Address Community-Level SDOH (N = 2858 Hospitals)

Measure	Hospitals with external partnerships, weighted % (95% CI)	
	To meet HRSNs	To meet SDOH (5)
≥1 Partnership type	68.0 (66.3-69.7)	53.7 (51.8-55.5)
Type of partnership		
Local or state public health departments	54.6 (52.8-56.5)	47.6 (45.8-49.4)
Health care practitioner outside the system	56.5 (54.6-58.3)	38.6 (36.8-40.4)
Other local, state, government, or social service organizations	54.0 (52.1-55.8)	40.7 (38.9-42.5)
Local organizations addressing food insecurity	52.3 (50.4-54.1)	37.4 (35.6-39.1)
Other community nonprofit organizations	50.5 (48.7-52.4)	38.6 (36.8-40.4)
Local organizations addressing transportation needs	52.8 (51.0-54.5)	27.7 (26.1-29.4)
Faith-based organizations	47.4 (45.6-49.2)	32.1 (30.4-33.8)
Law enforcement or safety forces	45.7 (43.9-47.5)	33.1 (31.4-34.8)
Kindergarten-12th grade schools	36.4 (34.6-38.2)	37.8 (36.0-39.5)
Local organizations addressing housing insecurity	47.0 (45.1-48.8)	25.8 (24.2-27.4)
Health care insurance outside the system	44.9 (43.0-46.7)	21.6 (20.1-23.1)
Local businesses or chambers of commerce	33.6 (31.9-35.3)	31.1 (29.4-32.8)
Colleges or universities	28.8 (27.1-30.5)	30.5 (28.8-32.2)
Local organizations providing legal assistance	35.1 (33.3-36.8)	13.3 (12.0-14.5)
Partnership types, mean (95% CI), No. (range, 0-14)	5.69 (5.50-5.88)	4.03 (3.85-4.20)

Abbreviations: HRSN, health-related social need; SDOH, social determinant of health.

### Screening Practices for HRSNs

Table 4 shows the results of ordinary least-square regression model analyses in which the outcome was the number of HRSNs screened. Hospitals reporting a higher number of HRSNs screened for were more likely to have active participation in accountable care contract programs (β = 1.03, SE = 0.13, *P* < .001) and bundled payment programs (β = 0.72, SE = 0.14, *P* < .001), teaching status (eg, major teaching vs nonteaching hospitals: β = 0.61; SE = 0.27; *P* < .001), and urbanicity (micropolitan vs metropolitan hospitals: β = 0.41; SE = 0.17; *P* = .01). Characteristics associated with hospitals that screened for fewer HRSNs included location in the West North Central (β = −0.60; SE = 0.25; *P* = .02) and Pacific (β = −0.53; SE = 0.27; *P* = .05) regions, compared with those in the West South Central region, and counties with higher percentages of uninsured adults aged younger than 65 years (β = −0.06; SE = 0.02; *P* = .001); rural location (vs metropolitan: β = −0.55; SE = 0.17; *P* = .04); and for-profit status (vs nonprofit: β = −0.91; SE = 0.17; *P* < .001) (Table 4). There were no significant associations between HRSN screening practices and hospital bed size, tax-exempt status (eg, government-owned), percentage of Medicaid patient discharges, area-level social disadvantage indicators, and other regional differences.

**Table 4.** Associations of Hospital Characteristics and Area-Level Social Disadvantage With HRSN Screening Practices, Strategies to Address HRSNs, and External Partnerships to Meet HRSNs and Community-Level Health Initiatives

Characteristic	No. of social needs screened		No. of social needs with interventions to address HRSNs		No. of external partnership types to meet HRSNs		No. of external partnership types to meet SDOH	
	β (SE)	*P* value	β (SE)	*P* value	β (SE)	*P* value	β (SE)	*P* value
Hospital size, beds								
<100	0 [Reference]	NA	0 [Reference]	NA	0 [Reference]	NA	0 [Reference]	NA
100-199	0.27 (0.17)	.10	0.08 (0.15)	.60	0.04 (0.28)	.90	0.44 (0.25)	.08
200-299	0.26 (0.21)	.21	0.35 (0.19)	.07	0.53 (0.36)	.14	0.11 (0.32)	.73
300-399	0.20 (0.26)	.45	0.10 (0.25)	.68	0.12 (0.45)	.80	0.50 (0.40)	.22
400-499	0.33 (0.33)	.31	0.19 (0.30)	.53	−0.06 (0.56)	.91	0.07 (0.50)	.88
≥500	0.36 (0.29)	.21	0.45 (0.27)	.09	0.64 (0.49)	.20	0.76 (0.43)	.08
Region								
West South Central	0 [Reference]	NA	0 [Reference]	NA	0 [Reference]	NA	0 [Reference]	NA
New England	−0.07 (0.36)	.85	0.42 (0.33)	.21	0.76 (0.61)	.22	1.30 (0.54)	.02
Mid-Atlantic	0.39 (0.29)	.18	0.49 (0.27)	.07	2.05 (0.50)	<.001	0.84 (0.44)	.06
South Atlantic	0.29 (0.21)	.17	0.31 (0.20)	.12	1.39 (0.37)	<.001	1.25 (0.33)	<.001
East North Central	−0.08 (0.25)	.74	0.07 (0.24)	.78	1.05 (0.44)	.02	0.37 (0.39)	.34
East South Central	0.37 (0.26)	.16	0.37 (0.24)	.12	0.91 (0.44)	.04	0.23 (0.39)	.56
West North Central	−0.60 (0.25)	.02	−0.32 (0.23)	.16	1.12 (0.42)	.008	−0.28 (0.37)	.45
Mountain	−0.44 (0.26)	.08	−0.25 (0.24)	.30	1.02 (0.44)	.02	0.09 (0.39)	.81
Pacific	−0.53 (0.27)	.05	0.29 (0.25)	.25	0.65 (0.46)	.16	0.08 (0.40)	.84
Teaching status								
Nonteaching	0 [Reference]	NA	0 [Reference]	NA	0 [Reference]	NA	0 [Reference]	NA
Other teaching	0.18 (0.17)	.31	0.44 (0.16)	.007	−0.37 (0.30)	.21	0.04 (0.27)	.88
Major teaching	0.61 (0.27)	.02	0.99 (0.25)	<.001	0.11 (0.46)	.82	0.07 (0.41)	.87
Control								
Nonprofit	0 [Reference]	NA	0 [Reference]	NA	0 [Reference]	NA	0 [Reference]	NA
Government-owned	−0.24 (0.15)	.10	−0.47 (0.14)	<.001	−0.45 (0.25)	.08	−0.90 (0.22)	<.001
For-profit	−0.91 (0.17)	<.001	−1.38 (0.16)	<.001	−1.12 (0.30)	<.001	−2.37 (0.26)	<.001
Urbanicity								
Metropolitan	0 [Reference]	NA	0 [Reference]	NA	0 [Reference]	NA	0 [Reference]	NA
Micropolitan	0.41 (0.17)	.01	0.27 (0.16)	.09	0.02 (0.29)	.93	0.49 (0.25)	.05
Rural	−0.55 (0.17)	.001	−0.48 (0.16)	.003	−0.18 (0.30)	.55	0.08 (0.26)	.75
Value-based care participation								
Accountable care contract	1.03 (0.13)	<.001	1.45 (0.12)	<.001	2.07 (0.23)	<.001	2.64 (0.20)	<.001
Bundled payment program	0.72 (0.14)	<.001	0.61 (0.13)	<.001	1.47 (0.24)	<.001	1.57 (0.21)	<.001
Proportion of Medicaid discharges	0.01 (0.01)	.26	0.00 (0.01)	.51	−0.01 (0.01)	.22	0.00 (0.01)	.65
County-level proportion of uninsured adults aged <65 y	−0.06 (0.02)	.001	−0.05 (0.02)	.003	0.04 (0.03)	.16	−0.03 (0.03)	.27
County-level Social Deprivation Index	0.00 (0.00)	.62	0.00 (0.00)	.26	0.00 (0.00)	.98	0.00 (0.00)	.31

Abbreviations: HRSN, health-related social need; NA, not applicable; SDOH, social determinant of health.

### Strategies and Programs to Address HRSNs

Similar to factors associated with screening practices, characteristics of hospitals reporting more strategies and programs to address HRSNs included having accountable care contract programs (β = 1.45; SE = 0.12; *P* < .001) and bundled payment programs (β = 0.61; SE = 0.13; *P* < .001) and major teaching (vs nonteaching: β = 0.99; SE = 0.25; *P* < .001) and minor teaching (vs nonteaching: β = 0.44; SE = 0.16; *P* = .007). Hospitals located in counties with higher percentages of uninsured adults aged younger than 65 years (β = −0.05; SE = 0.02; *P* = .003), for-profit status (vs nonprofit: β = −1.38; SE = 0.16; *P* < .001) and government-ownership (vs nonprofit: β = −0.47; SE = 0.14; *P* < .001), and rural location (vs metropolitan β = −0.48; SE = 0.16; *P* = .003) were less likely to have strategies or programs to address HRSNs (Table 4).

### HRSN-Directed External Partnership Types

Characteristics of hospitals reporting more HRSN-directed external partnership types include participation in accountable care contract programs (β = 2.07; SE = 0.23; *P* < .001) and bundled payment programs (β = 1.47; SE = 0.24; *P* < .001) and nonprofit status (vs for-profit: β = −1.12, SE = 0.30, *P* = .001) (Table 4). Hospitals located in the Mid-Atlantic (β = 2.05; SE = 0.50; *P* < .001), South Atlantic (β = 1.39; SE = 0.37; *P* < .001), East North Central (β = 1.05; SE = 0.44 *P* = .02), East South Central (β = 0.91; SE = 0.44; *P* = .04), West North Central (β = 1.12; SE = 0.42; *P* = .008), and Mountain (β = 1.02; SE = 0.44; *P* = .02) regions were more likely to have HRSN-directed external partnerships compared with those in New England, the Pacific, and West South Central regions (Table 4). We found no other significant associations.

### SDOH-Directed External Partnership Types

Similar to the HRSN analyses, hospitals were more likely to have SDOH-directed external partnerships if they were participating in accountable care contract programs (β = 2.64; SE = 0.20; *P* < .001) or bundled payment programs (β = 1.57; SE = 0.21; *P* < .001) or had nonprofit status (vs government-owned: β = −0.90; SE = 0.22; *P* < .001) or for-profit status (vs government-owned: β = −2.37; SE = 0.26; *P* < .001) (Table 4). We also found hospitals located in the New England (β = 1.30; SE = 0.54; *P* = .02) and South Atlantic (β = 1.25; SE = 0.33; *P* < .001) regions were more likely to have SDOH-directed external partnerships compared with those located in the West South Central. There were no other associations of hospital characteristics or area-level social disadvantage indicators with SHOH-directed external partnerships (Table 4).

For sensitivity analysis, we constructed alternative specifications of the regression and found the results to be robust to different cutoffs for number of HRSNs screened, without weighting, and found no joint associations of VBC and hospital characteristics, among other specifications, with outcomes of interest (eAppendix 2 in Supplement 1).

## Discussion

This cross-sectional study using nationwide data found that most hospitals reported conducting HRSN screenings and having strategies and programs to address patients’ multiple HRSNs and partnering with a diverse range of external organizations and sectors to address HRSNs and SDOH. Our findings support the idea that hospitals participating in VBC arrangements are particularly well-positioned to screen for and address multiple HRSNs for their patient populations, suggesting that population health management efforts may facilitate addressing social needs. Participating hospitals were also more likely to have community partners for patient referrals to attend to HRSNs beyond medical treatment and care. These relationships reinforce our understanding of health policies and the role of incentives, suggesting that there are identifiable health policy–related drivers that can contribute to overall progress in addressing HRSNs and SDOH.^24,25^ Although payers do not typically reimburse hospitals for the cost of screening, VBC programs, when successful, offer an alternative financing incentive for improving outcomes for patients with HRSNs, even when not directly intended to address equity.

In addition to VBC participation, the hospital characteristics most consistently associated with greater activity surrounding internal HRSN screening practices and strategies and programs to address patients’ HRSNs were nonprofit status and teaching status. In identifying these associations, our findings lend empirical credibility to the idea that addressing social needs and SDOH may be an extension of a hospital’s mission. The contribution by teaching hospitals and nonprofit hospitals informs the justification for the crucial policy support and financing that these hospitals receive. For example, nonprofit hospitals, which often cite health equity goals and address social disadvantages in their required community health needs assessments may be better positioned to screen and address HRSNs than other ownership types.^26,27^ Teaching hospitals serve a crucial role in providing standby services, subspecialty care, and other social benefits and have long financed the costs of those benefits even at a financial loss.^28^ Additionally, religious and teaching hospitals have their social missions alongside their clinical missions, and social needs screenings are sometimes part of their historical legacy.^26,29^

From a policy maker standpoint, the importance of these hospital characteristics underscores that there is a need for policy support in engaging in VBC arrangements. From a patient perspective, identifying hospitals with strategies to address social needs is a helpful indicator for the level of support from nonmedical services.^30^ These differences across hospital types in HRSN screening services may provide context for research on the need to socialize the use of *International Classification of Diseases, Ninth Revision, Clinical Modification *(*ICD-10-CM*) Z-codes used to capture and report social, economic and environmental factors known to affect health outcomes. The screening practices captured here may be a prerequisite to optimizing the alignment of screening tools with Z-codes.

Our findings regarding rural hospitals highlight some potential impediments to prioritizing identifying and addressing patients’ social needs^31,32,33^ amidst financial challenges, closures,^34^ and understaffing. Patients in rural settings face longstanding barriers to health care access, including the lack of public transportation and general lack of access to primary care. Policy makers should consider the results of this study when implementing equitable incentives and legislation to address rural patients’ social needs adequately.

While resource constraints may not prevent a large portion of hospitals from screening for at least 1 social need or having 1 partnership type, there was a significant decrease in prevalence for greater levels of screening or partnerships, leaving uncertain whether financial support or the differences in the opportunity to establish successful partnerships will be a barrier beyond the first phase of implementation. Our results point to the modifiable, policy-amenable and ready-to-use financial arrangements (ie, VBC arrangements) that are supportive of these screening activities.

### Limitations

This study has some limitations. Our findings are limited to the scope of the American Hospital Association survey instrument. Our results provide binary outcomes for each screening and partnership variable and do not measure the intensity or authenticity of engagement by the hospital, since the availability of a tool at a hospital does not guarantee that the tool will be used for all patients. Thus, our results identify which hospitals screen and for which social needs, but findings on whether screening applies to all patients or whether certain types are prioritized involves more nuanced future data collection. This type of data would elucidate the equitable distribution of screenings within hospitals, while our data do not indicate whether screening was equitably applied to all patients or whether certain types of HRSNs were prioritized. Scoping evidence suggests that most HRSN activities primarily occur in emergency departments during triage and intake.^35,36,37,38,39^ Although patients presenting with multiple social needs are more likely to use emergency departments and urgent care facilities,^18,40^ such localized efforts might grossly underestimate a hospital’s reported HRSN activity level overall if only a select subset of patient populations is being screened. Furthermore, the availability of a tool at a hospital does not guarantee the tool is being used for all patients. The research questions posed in this study may be further informed as new, more granular data become available. For example, Medicare’s new federally mandatory collection of screening data beginning in 2025 will require reporting what percentage of patients are screened for 5 HRSNs and what percentage had positive screening results.^41^

Our data interpretation is also limited in qualitatively understanding how patients are screened, what referrals are happening, and to what extent these fostered relationships mitigate patients’ inaccessibility to health care, experienced burdens, or negative health outcomes. This leaves unexplored the hospital relationships and barriers faced by social workers, community health workers, triage nurses, and other affiliated health care practitioners who often carry out HRSN-related activities.^42^

Future research should explore how strategies and partnerships vary by region, especially given our findings suggesting differences across urbanicity and geographical location. Additionally, this research primarily relies on self-reported survey data, which is subject to desirability bias.

## Conclusions

This cross-sectional study found that most US hospitals were screening patients for multiple HRSNs. Active participation in VBC, teaching hospital status, and nonprofit status were the characteristics most consistently associated with greater overall screening activities and number of related partnership types. As the US health care delivery system and reimbursement models continue to shift from fee-for-service payments to value-based payments, such policies and alternative payment models may portend long-term financial, social, and population health investment. Additionally, teaching hospitals and nonprofit hospitals continue to demonstrate leadership in screening for and addressing HRSNs. Achieving a culture of health equity will require the medical community to address multidimensional barriers and multilevel systemic inequities that contribute to poorer health and disease.^43,44,45^ Despite widespread screening and partnerships found in this study, the US lacks robust, universal social needs screening, and the extent of these efforts varies by hospital characteristics. These findings raise questions about what types of policy changes might be needed to support hospitals that implement programs benefiting SDOH drivers and patients with HRSNs. A multifaceted policy approach, including consistent and reliable funding of hospitals, is needed to identify and intervene to address the HRSNs.

## Notes

Supplement 1. **eAppendix 1.** Survey Instrument Details **eAppendix 2.** Sensitivity Analyses and Robustness

Supplement 2.
Data Sharing Statement
