# Supplementary material for: Screening for Health-Related Social Needs and Collaboration With External Partners Among US Hospitals
Source: JAMA Netw Open. 2023 Aug 23;6(8):e2330228. doi: 10.1001/jamanetworkopen.2023.30228 (PMC10448297; doi:10.1001/jamanetworkopen.2023.30228)
Supplement: Supplement 1. — eAppendix 1. Survey Instrument Details eAppendix 2. Sensitivity Analyses and Robustness [file jamanetwopen-e2330228-s001.pdf]

Supplemental Online Content

Ashe JJ, Baker MC, Alvarado CS, Alberti PM. Screening for health-related social needs and collaboration with external partners among US hospitals. *JAMA Netw Open*. 2023;6(8):e2330228. doi:10.1001/jamanetworkopen.2023.30228

eAppendix 1. Survey Instrument Details
eAppendix 2. Sensitivity Analyses and Robustness

This supplemental material has been provided by the authors to give readers additional information about their work.

© 2023 Ashe JJ et al. *JAMA Network Open.*

eAppendix 1. Survey Instrument Details

The ASDB asks each respondent two questions related to HRSNs "Which social needs of patients/social determinants of health in communities does your hospital or health system have programs or strategies to address?" and "Does your hospital or health system screen patients for social needs? If yes, please indicate which social needs are assessed." Eight HRSNs are provided as response options: housing (instability, quality, financing), food/hunger insecurity, transportation inaccessibility, utility needs, social isolation, interpersonal violence, employment and income, and education. We exclude health behaviors from the list of HRSN domains because although biobehavioral risk factors like substance use or physical inactivity contribute to biomedical risk and foretell of distal environmental inequities,[46] conflating individual health behaviors with HRSNs tends to engender inaccurate assessments of social and environmental risk factors.[47]

The ASDB also asks "Which types of organizations do you currently partner with in each of the following activities?" for two types of activities: (1) "Work together to meet patient social needs" and (2) "Work together to implement community-level initiatives to address social determinants of health" with 14 types of partners as response options: 1) health care providers outside of their respective system; 2) health insurance providers outside of their respective system; 3) local or state public health departments/organizations; 4) other local or state government agencies / social service organizations; 5) faith-based organizations; 6) local organizations addressing food insecurity; 7) local organizations addressing housing insecurity; 8) local organizations addressing transportation needs; 9) local organizations providing legal assistance; 10) community nonprofit organizations; 11) K-12 schools; 12) colleges/universities; 13) local businesses / chambers of commerce; and 14) law enforcement / safety forces.

© 2023 Ashe JJ et al. *JAMA Network Open.*

eAppendix 2. Sensitivity Analyses and Robustness

A series of *post-hoc* exploratory analyses were also conducted to measure the sensitivity of our core regression results, detailed below.

Alternative Measure of Screening Intensity

Instead of our core regression's use of the discrete number of HRSNs screened, each alternative specification in Supplemental Table 1 uses a logistic regression and replaces the original dependent variable with a binary variable for a specific cutoff for the number of HRSNs screened: one out of eight, four out of eight, and eight out of eight HRSNs.

Based on having odds ratios greater or less than 1, Supplemental Table 1 shows that the factors related to higher odds of screening various levels of HRSNs are similar to factors related to higher number of HRSNs screened in our original Table 3 results. Value-based care participation was consistently strongly related to our outcome. Major teaching status, non-profit status, and urbanicity were all positively related to our outcome, but to varying degrees depending on the model. Hospital size, while not related to number of HRSN screened/addressed in Table 3, was a significant predictor of whether a hospital screened for greater than zero HRSNs.

Inclusion of Hospital Financial Margins

© 2023 Ashe JJ et al. *JAMA Network Open.*

To measure the impact of financial health on screening, we specified models that included hospital financial margins as an additional covariate in the model. Because the data was not available for all hospitals, we limited the sample to only those hospitals participating in Medicare's Inpatient Prospective Payment System to ensure comparable financial data based on similar reporting protocols in our source data, Medicare cost reports.

Our first such model used a continuous measure of total all-payer margins and found no statistically significant relationship to HRSNs screening practices, strategies/programs, nor external partnerships to address either HRSNs or SDOH (*p*'s > .05). In our second model, we defined financial margins using three categories of all-payer patient care margins: greater than or equal to 1%, between 1% and -1%, or less than -1%, and similarly found no statistical significance of any category.

Exclusion of Rural Hospitals

To acknowledge that rural hospitals may experience other unique social, economic, and structural barriers related to HRSNs screening, we separately included a specification of the original regression that excluded rural hospitals from the sample. In analyses excluding rural hospitals, in addition to previously noted relations that remained, West North Central geographic locations to screening practices became statistically insignificant (p = .129); and higher SDI was related to more HRSN strategies/programs (p = .04).

Interactive Effects

© 2023 Ashe JJ et al. *JAMA Network Open.*

Because of the large correlations with value-based care found in our original regression, we examined the potential joint effects of hospital's participation in accountable care contracts, bundled payment programs, and teaching affiliation status by creating indicator variables to reflect differences across these three factors, whereby the reference group for regression models were nonteaching hospitals not participating in either accountable care contracts or bundled payment programs. In these analyses only, major teaching and other teaching hospitals were combined to reflect any teaching status. Analyses that sought to explore distinctions across hospital's participation in accountable care contracts, bundled payment programs, and teaching affiliation status confirmed a main effect of teaching status across all outcome variables, but a clearer difference emerged across nonteaching and teaching hospitals that participated in both accountable care contracts and bundled payment programs compared to hospitals of less involvement. Among these hospitals, screening rates and activities related to addressing HRSNs and SDOH (strategies/programs, external partnerships) were highest, irrespective of teaching affiliation (p's < .05).

Unweighted Results

We repeated original regression results from Table 3, but modified the model to exclude any weighting variable, in contrast to Table 3's use of a weighting variable to adjust for non-response bias. The results from this unweighted model in Supplemental Table 2 show very similar results to Table 3, with some small differences in statistical significance but no change to the overall picture. The largest difference between the weighted an unweighted model is for select bed size and region categories which were statistically significantly different only before weighting.

© 2023 Ashe JJ et al. *JAMA Network Open.*

Supplemental Table 1. Logistic regression analyses assessing influences of hospital characteristics and area-level social disadvantage to HRSNs screening practices, strategies to address HRSNs, and hospitals' external partnerships to meet HRSNs and community-level health initiatives

	**Model 5**		**Model 6**		**Model 7**	
Outcome	Whether Hospitals Screened for ≥ 1 HRSN		Whether Hospitals Screened for ≥ 4 of the 8 HRSNs		Whether Hospitals Screened for all eight HRSNs	
**Hospital Characteristics**	*OR* (CI)	*p* value	*OR* (CI)	*p* value	*OR* (CI)	*p* value
Hospital bed size						
<100 beds [reference group = 0]						
100 - 199 beds	1.51 (1.18-1.94)**	.001	1.31 (1.07-1.60)**	.009	0.87 (0.70-1.07)	.18
200 - 299 beds	2.09 (1.47-2.95)***	<.001	1.38 (1.06-1.80)*	.02	0.70 (0.53-0.91)**	.008
300 - 399 beds	1.52 (0.99-2.35)	.06	1.11 (0.79-1.54)	.56	0.89 (0.64-1.22)	.46
400 - 499 beds	2.30 (1.28-4.13)**	.006	1.38 (0.90-2.12)	.14	0.95 (0.64-1.40)	.78
500 ≤ beds	2.22 (1.33-3.70)**	.002	1.59 (1.08-2.35)*	.02	0.87 (0.61-1.23)	.44
Region						
West South Central [reference group = 0]						
New England	1.08 (0.60-1.96)	.79	1.06 (0.68-1.67)	.79	1.26 (0.80-2.00)	.32
Mid-Atlantic	1.16 (0.71-1.90)	.55	1.17 (0.80-1.71)	.41	3.17 (2.18-4.60)***	<.001
South Atlantic	0.86 (0.64-1.17)	.33	1.24 (0.95-1.60)	.11	1.92 (1.44-2.54)***	<.001
East North Central	0.92 (0.63-1.35)	.68	0.93 (0.68-1.26)	.63	1.65 (1.18-2.31)**	.003
East South Central	1.66 (1.13-2.43)*	.01	1.26 (0.92-1.72)	.15	1.73 (1.22-2.43)**	.002

© 2023 Ashe JJ et al. *JAMA Network Open.*

West North Central	0.64 (0.46-0.90)*	.01	0.62 (0.47-0.84)**	.002	1.04 (0.74-1.46)	.83
Mountain	0.50 (0.35-0.70)***	<.001	0.69 (0.51-0.94)*	.02	1.52 (1.10-2.13)*	.01
Pacific	0.68 (0.47-1.00)*	.05	0.72 (0.52-0.99)*	.04	1.06 (0.74-1.53)	.75
Teaching status						
Non-teaching [reference group = 0]						
Other teaching	0.77 (0.58-1.03)	.07	1.15 (0.92-1.44)	.22	1.21 (0.97-1.50)	.09
Major teaching	1.04 (0.64-1.70)	.87	1.48 (1.03-2.15)*	.04	1.67 (1.21-2.30)**	.002
Control						
Non-profit [reference group = 0]						
Government-owned	0.82 (0.67-1.00)	.05	0.93 (0.78-1.11)	.41	0.95 (0.78-1.16)	.62
For-profit	0.49 (0.39-0.62)***	<.001	0.57 (0.47-0.70)***	<.001	0.82 (0.65-1.03)	.08
Urbanicity						
Metropolitan [reference group = 0]						
Micropolitan	1.30 (1.02-1.67)*	.04	1.39 (1.14-1.71)**	.002	1.22 (0.99-1.50)	.07
Rural	0.73 (0.58-0.92)**	.007	0.81 (0.66-0.99)*	.04	0.74 (0.58-0.93)**	.009
Value-based care participation						
Hospitals reporting an accountable care contract	2.58 (02.02-3.29)***	<.001	1.84 (1.55-2.19)***	<.001	2.06 (1.75-2.42)***	<.001
Hospitals reporting a bundled payment programs	1.44 (1.14-1.81)**	.002	1.67 (1.40-1.99)***	<.001	1.42 (1.20-1.68)***	<.001
Other characteristics						

© 2023 Ashe JJ et al. *JAMA Network Open.*

Hospitals' % of Medicaid discharges	1.01 (1.00-1.02)*	.04	1.00 (1.00-1.01)	.54	1.00 (1.00-1.01)*	.04
Hospitals' county location % of uninsured adults < 65 years old	0.96 (0.93-0.98)***	<.001	0.96 (0.94-0.98)**	<.001	1.00 (0.97-1.02)	.73
Hospitals' county location Social Deprivation Index	1.00 (1.00-1.00)	.80	1.00 (1.00-1.00)	.48	1.00 (1.00-1.00)	.98

Source. Authors' analysis of American Hospital Association Annual Survey Database data (2020), County Health Rankings (2020), and Robert Graham Center (2015 - 2019). **Note.** The reference groups noted in each category were selected based on being the largest subgroupings. Regression coefficients represent weight adjusted analyses.
*p < .05; **p < .01; ***p < .001

Supplemental Table 2. Unweighted regression analyses assessing influences of hospital characteristics and area-level social disadvantage to HRSNs screening practices, strategies to address HRSNs, and hospitals' external partnerships to meet HRSNs and community-level health initiatives

	Model 1		Model 2		Model 3		Model 4	
Outcome	Number of Social Needs Screened		Number of Social Needs with Interventions to Address HRSNs		Number of External Partnership Types to meet HRSNs		Number of External Partnership Types to meet SDOH	
**Hospital Characteristics**	*B* (SE)	*p* value	*B* (SE)	*p* value	*B* (SE)	*p* value	*B* (SE)	*p* value
Hospital bed size								
<100 beds [reference group = 0]								
100 - 199 beds	0.34 (0.17)*	.04	0.15 (0.16)	.35	0.09 (0.29)	.76	0.56 (0.26)*	.03
200 - 299 beds	0.50 (0.21)*	.02	0.50 (0.19)*	.01	0.49 (0.36)	.18	0.24 (0.33)	.45
300 - 399 beds	0.30 (0.25)	.22	0.22 (0.23)	.35	0.06 (0.44)	.88	0.55 (0.39)	.16

© 2023 Ashe JJ et al. *JAMA Network Open.*

400 - 499 beds	0.41 (0.30)	.18	0.21 (0.28)	.45	-0.09 (0.54)	.87	0.13 (0.48)	.78
500 ≤ beds	0.50 (0.26)	.06	0.57 (0.24)*	.02	0.64 (0.46)	.16	0.75 (0.431)	.07
Region								
West South Central [reference group = 0]								
New England	-0.03 (0.33)	.93	0.58 (0.31)	.07	0.45 (0.59)	.44	1.40 (0.53)*	.01
Mid-Atlantic	0.42 (0.27)	.12	0.63 (0.25)*	.01	1.83 (0.48)***	<.001	0.85 (0.43)*	.05
South Atlantic	0.23 (0.21)	.27	0.31 (0.20)	.11	1.18 (0.37)**	<001	1.30 (0.33)***	<.001
East North Central	-0.07 (0.24)	.77	0.21 (0.22)	.34	0.68 (0.41)	.10	0.38 (0.37)	.31
East South Central	0.17 (0.31)	.58	0.45 (0.29)	.11	0.39 (0.54)*	.47	0.10 (0.48)	.84
West North Central	-0.47 (0.23)*	.04	-0.12 (0.22)	.57	0.95 (0.40)*	.02	-0.27 (0.36)	.44
Mountain	-0.24 (0.25)	.34	0.05 (0.23)	.84	0.97 (0.44)*	.03	0.29 (0.40)	.47
Pacific	-0.40 (0.26)	.13	0.51 (0.25)*	.04	0.45 (0.46)	.33	0.05 (0.42)	.90
Teaching status								
Non-teaching [reference group = 0]								
Other teaching	0.11 (0.17)	.51	0.36 (0.16)*	.02	-0.27 (0.29)	.36	0.04 (0.26)	.87
Major teaching	0.61 (0.25)*	.01	0.91 (0.23)**	.001	0.09 (0.44)	.84	0.10 (0.37)	.80
Control								
Non-profit [reference group = 0]								
Government-owned	-0.30 (0.15)*	.05	-0.38 (0.14)**	.007	-0.37 (0.26)	.16	-0.81 (0.24)***	<.001

© 2023 Ashe JJ et al. *JAMA Network Open.*

For-profit	-0.96 (0.20)***	<.001	-1.31 (0.19)***	<.001	-1.22 (0.35)***	<.001	-2.28 (0.32)***	<.001
Urbanicity								
Metropolitan [reference group = 0]								
Micropolitan	0.36 (0.16)*	.03	0.21 (0.15)	.16	0.11 (0.29)	.71	0.35 (0.25)	.17
Rural	-0.70 (0.18)***	<.001	-0.62 (0.17)***	<.001	-0.16 (0.31)	.61	-0.15 (0.28)	.60
Value-based care participation								
Hospitals reporting an accountable care contract	0.98 (0.13)***	<.001	1.32 (0.12)***	<.001	2.10 (0.22)***	<.001	2.54 (0.20)***	<.001
Hospitals reporting a bundled payment programs	0.69 (0.13)***	<.001	0.65 (0.12)***	<.001	1.49 (0.23)***	<.001	1.63 (0.21)***	<.001
Other characteristics								
Hospitals' % of Medicaid discharges	0.00 (0.01)	.26	0.00 (0.01)	.42	-0.01 (0.01)	.25	0.00 (0.01)	.71
Hospitals' county location % of uninsured adults < 65 years old	-0.05 (0.02)**	.005	-0.04 (0.02)*	.01	0.01 (0.03)	.71	-0.03 (0.03)	.31
Hospitals' county location Social Deprivation Index	0.00 (0.00)	.62	0.00 (0.00)	.17	0.00 (0.00)	.66	-0.01 (0.00)	.11

© 2023 Ashe JJ et al. *JAMA Network Open.*

Source Authors' analysis of American Hospital Association Annual Survey Database data (2020), County Health Rankings (2020), and Robert Graham Center (2015 - 2019). **Note.** The reference groups noted in each category were selected based on being the largest subgroupings. Regression coefficients represent weight adjusted analyses.

*$p < .05$; **$p < .01$; ***$p < .001$

© 2023 Ashe JJ et al. *JAMA Network Open.*
