# Supplementary material for: Screening for Health-Related Social Needs and Collaboration With External Partners Among US Hospitals
Source: JAMA Netw Open. 2023 Aug 23;6(8):e2330228. doi: 10.1001/jamanetworkopen.2023.30228 (PMC10448297; doi:10.1001/jamanetworkopen.2023.30228)
Supplement: Supplement 2. — Data Sharing Statement [file jamanetwopen-e2330228-s002.pdf]

Data Sharing Statement

Ashe. Screening for Health-Related Social Needs and Collaboration With External Partners Among US Hospitals. *JAMA Netw Open*. Published August 22, 2023. doi:10.1001/jamanetworkopen.2023.30228

Data
Data available: No
